# 3-(2,4-Dichloro­phen­yl)-5-(4-fluoro­phen­yl)-2-methyl-7-(trifluoro­meth­yl)pyrazolo­[1,5-*a*]pyrimidine

**DOI:** 10.1107/S1600536812017345

**Published:** 2012-04-25

**Authors:** Ju Liu, Zhi-Qiang Cai, Yang Wang, Ming-Jun Jiang, Li-Feng Xu

**Affiliations:** a College of Pharmacy, Liaoning University, Shenyang 110036, People’s Republic of China; b Panjin Vocational and Technical College, Panjin 120010, People’s Republic of China; c Tianjin Key Laboratory of Molecular Design and Drug Discovery, State Key Laboratory of Drug Delivery Technology and Pharmacokinetics, Tianjin Institute of Pharmaceutical Research, Tianjin 300193, People’s Republic of China

## Abstract

In the title compound, C_20_H_11_Cl_2_F_4_N_3_, the central pyrazolo­[1,5-*a*]pyrimidine unit is almost planar [the mean deviation from the best least-square plane through the nine atoms is 0.006 (2) Å]. The fluoro­benzene ring is rotated out of this plane by 10.3 (3)°, whereas the dichloro­benzene ring is rotated by 46.2 (3)°. The crystal packing is dominated by Cl⋯Cl inter­actions of 3.475 (3) Å and van der Waals inter­actions.

## Related literature

For the synthesis of other pyrazolo­[1,5-*a*]pyrimidine derivatives and for their pharmacological applications, see: Fraley *et al.* (2012 ▶); Novinson *et al.* (1976 ▶); Senga *et al.* (1981 ▶); Suzuki *et al.* (2001 ▶). For related structures, see: Liu *et al.* (2012 ▶); Bui *et al.* (2009 ▶).
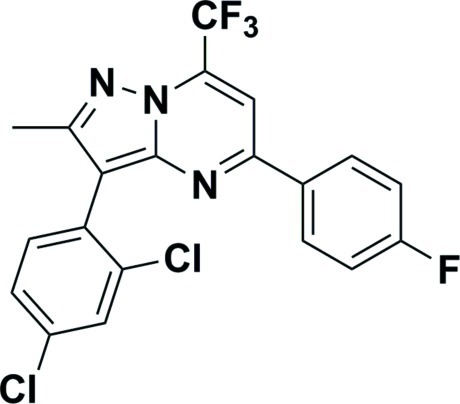


## Experimental

### 

#### Crystal data

C_20_H_11_Cl_2_F_4_N_3_
*M* *_r_* = 440.22
Orthorhombic, 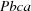

*a* = 9.5361 (19) Å
*b* = 15.941 (3) Å
*c* = 24.853 (5) Å
*V* = 3778.0 (13) Å^3^
*Z* = 8
Mo *K*α radiation
μ = 0.39 mm^−1^
*T* = 298 K
0.20 × 0.18 × 0.16 mm

#### Data collection

Rigaku Saturn diffractometer
Absorption correction: multi-scan (*CrystalClear*; Rigaku/MSC, 2005) ▶ *T*_min_ = 0.926, *T*_max_ = 0.940
34334 measured reflections
4477 independent reflections
3517 reflections with *I* > 2σ(*I*)
*R*_int_ = 0.049

#### Refinement

*R*[*F* ^2^ > 2σ(*F* ^2^)] = 0.055
*wR*(*F* ^2^) = 0.153
*S* = 1.08
4477 reflections
264 parameters
H-atom parameters constrained
Δρ_max_ = 0.27 e Å^−3^
Δρ_min_ = −0.34 e Å^−3^

### 

Data collection: *RAPID-AUTO* (Rigaku, 1998) ▶; cell refinement: *RAPID-AUTO* ▶; data reduction: *CrystalClear* (Rigaku/MSC, 2005) ▶; program(s) used to solve structure: *SHELXS97* (Sheldrick, 2008 ▶); program(s) used to refine structure: *SHELXL97* (Sheldrick, 2008 ▶); molecular graphics: *SHELXTL* (Sheldrick, 2008 ▶); software used to prepare material for publication: *SHELXTL*.

## Supplementary Material

Crystal structure: contains datablock(s) I, global. DOI: 10.1107/S1600536812017345/kp2407sup1.cif

Structure factors: contains datablock(s) I. DOI: 10.1107/S1600536812017345/kp2407Isup2.hkl

Supplementary material file. DOI: 10.1107/S1600536812017345/kp2407Isup3.cml

Additional supplementary materials:  crystallographic information; 3D view; checkCIF report

## Figures and Tables

**Fig. 1.**
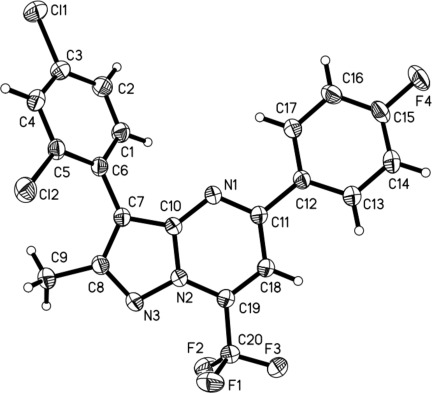
The structure of C20H11Cl2F4N3 with all non-H atom-labelling scheme and ellipsoids drawn at the 50% probability level.

## supplementary crystallographic information

### Comment

Pyrazolo[1,5-*a*]pyrimidines are purine analogues with useful properties as antimetabolites in purine biochemical reactions. They are used in a wide array of synthetic and medical chemistry, such as antitrypanosomal and antischistosomal activities, KDR kinase inhibitors, selective peripheral benzodiazepine receptor ligands (Fraley *et al.*, 2012). We have adapted the known method and synthesised the compound. The new molecule is expected to exhibit enhanced biological activity. To characterize our product, its single-crystal structure was determined.

The title molecule (Fig. 1) bond lengths and angles are generally within normal ranges. The dihedral angles formed by the two benzene rings is 50.76 (3) °. The relatively small deviations from the ring planes of the attached substituents are defined by corresponding torsion angles: C(11)—C(18)—C(19)—C(20) of -176.8 (2) for the threefluromethyl group (C20); N(2)—N(3)—C(8)—C(9) of 177.5 (2) for methyl group (C9); C(13)—C(14)—C(15)—F(4) of -179.7 (2) for F4, and Cl(1)—C(3)—C(4)—C(5) of 179.2 (2) for Cl1; Cl(2)—C(5)—C(6)—C(1) of -174.8 (2)°. The crystal structure is held together by van der Waals forces and pronounced Cl···Cl interaction of 3.475()Å (Bui *et al.*, 2009).

### Experimental

A mixture of the corresponding 4-(2,4-dichlorophenyl)-3-methyl-1*H*-pyrazol -5-amine (1.40 g, 5.78 mmol) and the 4,4,4-trifluoro-1-(4-fluorophenyl) butane-1,3-dione (1.49 g, 6.36 mmol) in a flask (25 mL) was heated at 433-438 K for 2.5 h, allowing elimination of the water evolved. After cooling to room temperature, the solid in the flask was recrystallised from methanol to yield the title compound as a yellow solid (1.70 g, 66.78%). Crystals suitable for X-ray analysis were obtained from a mixture of solvents ethanol/acetone(1:1) by slow evaporation.

### Refinement

All H atoms were geometrically positioned (C—H 0.93–0.98 Å) and treated as riding, with *U*_iso_(H) = 1.2Ueq(C).Due to lack of heavy atoms, Friedel pairs were merged in refinement.

### Crystal data

**Table:**

C_20_H_11_Cl_2_F_4_N_3_	*F*(000) = 1776
*M**_r_* = 440.22	*D*_x_ = 1.548 Mg m^−^^3^
Orthorhombic, *P**b**c**a*	Mo *K*α radiation, λ = 0.71073 Å
Hall symbol: -P 2ac 2ab	Cell parameters from 8300 reflections
*a* = 9.5361 (19) Å	θ = 2.3–27.9°
*b* = 15.941 (3) Å	µ = 0.39 mm^−^^1^
*c* = 24.853 (5) Å	*T* = 298 K
*V* = 3778.0 (13) Å^3^	Block, yellow
*Z* = 8	0.20 × 0.18 × 0.16 mm

### Data collection

**Table:**

Rigaku Saturn diffractometer	4477 independent reflections
Radiation source: rotating anode	3517 reflections with *I* > 2σ(*I*)
Confocal monochromator	*R*_int_ = 0.049
ω scans	θ_max_ = 27.9°, θ_min_ = 2.6°
Absorption correction: multi-scan (*CrystalClear*; Rigaku/MSC, 2005)	*h* = −11→12
*T*_min_ = 0.926, *T*_max_ = 0.940	*k* = −20→20
34334 measured reflections	*l* = −32→32

### Refinement

**Table:**

Refinement on *F*^2^	Secondary atom site location: difference Fourier map
Least-squares matrix: full	Hydrogen site location: inferred from neighbouring sites
*R*[*F*^2^ > 2σ(*F*^2^)] = 0.055	H-atom parameters constrained
*wR*(*F*^2^) = 0.153	*w* = 1/[σ^2^(*F*_o_^2^) + (0.0762*P*)^2^ + 0.9456*P*] where *P* = (*F*_o_^2^ + 2*F*_c_^2^)/3
*S* = 1.08	(Δ/σ)_max_ = 0.002
4477 reflections	Δρ_max_ = 0.27 e Å^−^^3^
264 parameters	Δρ_min_ = −0.34 e Å^−^^3^
0 restraints	Extinction correction: *SHELXL97* (Sheldrick, 2008), Fc^*^=kFc[1+0.001xFc^2^λ^3^/sin(2θ)]^-1/4^
Primary atom site location: structure-invariant direct methods	Extinction coefficient: 0.0093 (10)

### Special details

**Table:**

Geometry. All e.s.d.'s (except the e.s.d. in the dihedral angle between two l.s. planes) are estimated using the full covariance matrix. The cell e.s.d.'s are taken into account individually in the estimation of e.s.d.'s in distances, angles and torsion angles; correlations between e.s.d.'s in cell parameters are only used when they are defined by crystal symmetry. An approximate (isotropic) treatment of cell e.s.d.'s is used for estimating e.s.d.'s involving l.s. planes.
Refinement. Refinement of *F*^2^ against ALL reflections. The weighted *R*-factor *wR* and goodness of fit *S* are based on *F*^2^, conventional *R*-factors *R* are based on *F*, with *F* set to zero for negative *F*^2^. The threshold expression of *F*^2^ > σ(*F*^2^) is used only for calculating *R*-factors(gt) *etc*. and is not relevant to the choice of reflections for refinement. *R*-factors based on *F*^2^ are statistically about twice as large as those based on *F*, and *R*- factors based on ALL data will be even larger.

### Fractional atomic coordinates and isotropic or equivalent isotropic displacement parameters (Å^2^)

**Table:**

	*x*	*y*	*z*	*U*_iso_*/*U*_eq_	
Cl1	0.65368 (10)	−0.11103 (5)	0.54765 (3)	0.0819 (3)	
Cl2	0.61073 (8)	0.22387 (4)	0.56049 (3)	0.0626 (2)	
F1	0.25116 (18)	0.44152 (9)	0.75453 (7)	0.0707 (4)	
F2	0.06282 (16)	0.37077 (10)	0.75226 (7)	0.0726 (5)	
F3	0.1688 (2)	0.39016 (11)	0.82698 (6)	0.0841 (6)	
F4	0.6356 (2)	−0.01913 (11)	0.96188 (6)	0.0839 (5)	
N1	0.44109 (19)	0.15634 (11)	0.74870 (6)	0.0430 (4)	
N2	0.30004 (19)	0.27037 (11)	0.71649 (6)	0.0416 (4)	
N3	0.2542 (2)	0.30511 (11)	0.66969 (7)	0.0467 (4)	
C1	0.4349 (2)	0.03787 (14)	0.64421 (9)	0.0495 (5)	
H1	0.3749	0.0312	0.6734	0.059*	
C2	0.4924 (3)	−0.03226 (14)	0.62119 (10)	0.0557 (6)	
H2	0.4712	−0.0854	0.6343	0.067*	
C3	0.5822 (3)	−0.02252 (15)	0.57820 (9)	0.0540 (6)	
C4	0.6166 (3)	0.05579 (15)	0.55899 (9)	0.0534 (6)	
H4	0.6785	0.0617	0.5303	0.064*	
C5	0.5576 (2)	0.12577 (13)	0.58318 (8)	0.0442 (5)	
C6	0.4625 (2)	0.11903 (13)	0.62578 (8)	0.0409 (5)	
C7	0.3930 (2)	0.19044 (13)	0.65241 (8)	0.0414 (5)	
C8	0.3106 (2)	0.25618 (13)	0.63129 (8)	0.0440 (5)	
C9	0.2755 (3)	0.27527 (16)	0.57386 (8)	0.0575 (6)	
H9A	0.1841	0.3003	0.5720	0.086*	
H9B	0.2763	0.2243	0.5533	0.086*	
H9C	0.3437	0.3135	0.5595	0.086*	
C10	0.3844 (2)	0.20017 (13)	0.70785 (8)	0.0407 (4)	
C11	0.4124 (2)	0.18080 (13)	0.79856 (8)	0.0417 (5)	
C12	0.4725 (2)	0.12953 (13)	0.84255 (8)	0.0422 (5)	
C13	0.4680 (3)	0.15520 (15)	0.89601 (9)	0.0566 (6)	
H13	0.4272	0.2064	0.9048	0.068*	
C14	0.5237 (3)	0.10527 (17)	0.93628 (9)	0.0644 (7)	
H14	0.5217	0.1227	0.9720	0.077*	
C15	0.5813 (3)	0.03045 (15)	0.92273 (9)	0.0557 (6)	
C16	0.5893 (3)	0.00274 (14)	0.87055 (10)	0.0562 (6)	
H16	0.6305	−0.0486	0.8624	0.067*	
C17	0.5345 (3)	0.05311 (14)	0.83055 (9)	0.0499 (5)	
H17	0.5392	0.0355	0.7949	0.060*	
C18	0.3272 (2)	0.25229 (13)	0.80901 (8)	0.0448 (5)	
H18	0.3090	0.2685	0.8443	0.054*	
C19	0.2727 (2)	0.29661 (13)	0.76758 (8)	0.0428 (5)	
C20	0.1884 (3)	0.37488 (14)	0.77517 (9)	0.0499 (5)	

### Atomic displacement parameters (Å^2^)

**Table:**

	*U*^11^	*U*^22^	*U*^33^	*U*^12^	*U*^13^	*U*^23^
Cl1	0.1165 (7)	0.0593 (4)	0.0700 (5)	0.0266 (4)	0.0131 (4)	−0.0112 (3)
Cl2	0.0768 (5)	0.0527 (4)	0.0582 (4)	−0.0109 (3)	0.0173 (3)	0.0097 (3)
F1	0.0777 (10)	0.0447 (8)	0.0896 (11)	0.0045 (7)	0.0037 (8)	0.0058 (7)
F2	0.0491 (9)	0.0751 (10)	0.0938 (12)	0.0153 (7)	−0.0084 (8)	−0.0075 (9)
F3	0.1183 (15)	0.0839 (11)	0.0501 (9)	0.0531 (10)	0.0102 (8)	−0.0051 (7)
F4	0.1151 (14)	0.0751 (11)	0.0615 (9)	0.0177 (10)	−0.0265 (9)	0.0221 (8)
N1	0.0519 (10)	0.0432 (9)	0.0341 (8)	0.0063 (8)	0.0011 (7)	0.0031 (7)
N2	0.0484 (10)	0.0413 (9)	0.0350 (8)	0.0070 (7)	−0.0006 (7)	0.0019 (7)
N3	0.0564 (11)	0.0457 (10)	0.0382 (9)	0.0073 (8)	−0.0067 (8)	0.0046 (7)
C1	0.0567 (14)	0.0476 (12)	0.0442 (11)	−0.0017 (10)	0.0107 (10)	0.0050 (9)
C2	0.0717 (16)	0.0405 (11)	0.0549 (13)	−0.0022 (11)	0.0065 (11)	0.0046 (10)
C3	0.0676 (15)	0.0467 (12)	0.0476 (12)	0.0093 (11)	0.0010 (11)	−0.0059 (10)
C4	0.0631 (15)	0.0569 (14)	0.0403 (11)	0.0036 (11)	0.0107 (10)	−0.0007 (9)
C5	0.0525 (13)	0.0451 (11)	0.0352 (10)	−0.0032 (9)	0.0033 (9)	0.0032 (8)
C6	0.0475 (12)	0.0417 (10)	0.0335 (9)	−0.0014 (9)	−0.0009 (8)	−0.0003 (8)
C7	0.0485 (12)	0.0413 (11)	0.0345 (9)	0.0007 (9)	0.0022 (8)	0.0017 (8)
C8	0.0518 (13)	0.0428 (11)	0.0374 (10)	−0.0006 (9)	−0.0029 (8)	0.0024 (8)
C9	0.0748 (17)	0.0587 (14)	0.0389 (11)	0.0084 (12)	−0.0087 (11)	0.0016 (10)
C10	0.0463 (11)	0.0394 (10)	0.0364 (10)	0.0042 (8)	0.0017 (8)	0.0035 (8)
C11	0.0481 (12)	0.0412 (11)	0.0357 (10)	0.0035 (9)	0.0029 (8)	0.0023 (8)
C12	0.0501 (12)	0.0402 (10)	0.0363 (10)	0.0005 (9)	−0.0005 (8)	0.0034 (8)
C13	0.0766 (17)	0.0541 (13)	0.0392 (11)	0.0139 (12)	0.0002 (11)	0.0021 (10)
C14	0.089 (2)	0.0681 (16)	0.0360 (11)	0.0103 (14)	−0.0060 (12)	0.0032 (10)
C15	0.0661 (15)	0.0544 (13)	0.0465 (12)	0.0013 (11)	−0.0125 (11)	0.0155 (10)
C16	0.0677 (16)	0.0443 (12)	0.0567 (14)	0.0089 (11)	−0.0113 (11)	0.0048 (10)
C17	0.0630 (15)	0.0459 (12)	0.0409 (11)	0.0067 (10)	−0.0064 (10)	0.0000 (9)
C18	0.0529 (13)	0.0452 (11)	0.0362 (10)	0.0071 (9)	0.0056 (9)	0.0022 (8)
C19	0.0463 (12)	0.0432 (11)	0.0388 (10)	0.0033 (9)	0.0033 (8)	0.0009 (8)
C20	0.0559 (14)	0.0485 (12)	0.0453 (12)	0.0114 (10)	0.0021 (10)	0.0000 (9)

### Geometric parameters (Å, º)

**Table:**

Cl1—C3	1.741 (2)	C7—C10	1.389 (3)
Cl2—C5	1.738 (2)	C7—C8	1.411 (3)
F1—C20	1.323 (3)	C8—C9	1.497 (3)
F2—C20	1.328 (3)	C9—H9A	0.9600
F3—C20	1.324 (3)	C9—H9B	0.9600
F4—C15	1.356 (3)	C9—H9C	0.9600
N1—C11	1.327 (2)	C11—C18	1.424 (3)
N1—C10	1.346 (2)	C11—C12	1.480 (3)
N2—N3	1.360 (2)	C12—C17	1.386 (3)
N2—C19	1.362 (2)	C12—C13	1.391 (3)
N2—C10	1.395 (3)	C13—C14	1.385 (3)
N3—C8	1.345 (3)	C13—H13	0.9300
C1—C2	1.370 (3)	C14—C15	1.355 (4)
C1—C6	1.397 (3)	C14—H14	0.9300
C1—H1	0.9300	C15—C16	1.372 (3)
C2—C3	1.378 (3)	C16—C17	1.381 (3)
C2—H2	0.9300	C16—H16	0.9300
C3—C4	1.376 (3)	C17—H17	0.9300
C4—C5	1.387 (3)	C18—C19	1.353 (3)
C4—H4	0.9300	C18—H18	0.9300
C5—C6	1.398 (3)	C19—C20	1.496 (3)
C6—C7	1.474 (3)		
			
C11—N1—C10	117.98 (18)	N1—C10—N2	122.14 (18)
N3—N2—C19	127.64 (17)	C7—C10—N2	106.04 (17)
N3—N2—C10	112.34 (16)	N1—C11—C18	121.53 (18)
C19—N2—C10	120.02 (17)	N1—C11—C12	116.59 (18)
C8—N3—N2	104.03 (16)	C18—C11—C12	121.88 (18)
C2—C1—C6	122.9 (2)	C17—C12—C13	118.5 (2)
C2—C1—H1	118.6	C17—C12—C11	119.43 (19)
C6—C1—H1	118.6	C13—C12—C11	122.08 (19)
C1—C2—C3	118.7 (2)	C14—C13—C12	120.6 (2)
C1—C2—H2	120.6	C14—C13—H13	119.7
C3—C2—H2	120.6	C12—C13—H13	119.7
C4—C3—C2	121.3 (2)	C15—C14—C13	118.8 (2)
C4—C3—Cl1	119.35 (19)	C15—C14—H14	120.6
C2—C3—Cl1	119.35 (19)	C13—C14—H14	120.6
C3—C4—C5	118.8 (2)	C14—C15—F4	119.3 (2)
C3—C4—H4	120.6	C14—C15—C16	122.7 (2)
C5—C4—H4	120.6	F4—C15—C16	118.0 (2)
C4—C5—C6	122.00 (19)	C15—C16—C17	118.2 (2)
C4—C5—Cl2	117.68 (16)	C15—C16—H16	120.9
C6—C5—Cl2	120.28 (16)	C17—C16—H16	120.9
C1—C6—C5	116.20 (19)	C16—C17—C12	121.2 (2)
C1—C6—C7	118.89 (18)	C16—C17—H17	119.4
C5—C6—C7	124.89 (18)	C12—C17—H17	119.4
C10—C7—C8	104.67 (18)	C19—C18—C11	119.89 (19)
C10—C7—C6	123.92 (18)	C19—C18—H18	120.1
C8—C7—C6	131.06 (18)	C11—C18—H18	120.1
N3—C8—C7	112.92 (18)	C18—C19—N2	118.42 (19)
N3—C8—C9	118.01 (19)	C18—C19—C20	123.09 (19)
C7—C8—C9	129.0 (2)	N2—C19—C20	118.45 (18)
C8—C9—H9A	109.5	F1—C20—F3	107.1 (2)
C8—C9—H9B	109.5	F1—C20—F2	106.34 (19)
H9A—C9—H9B	109.5	F3—C20—F2	107.4 (2)
C8—C9—H9C	109.5	F1—C20—C19	112.21 (19)
H9A—C9—H9C	109.5	F3—C20—C19	110.62 (18)
H9B—C9—H9C	109.5	F2—C20—C19	112.9 (2)
N1—C10—C7	131.82 (19)		
			
C19—N2—N3—C8	179.8 (2)	N3—N2—C10—C7	0.4 (2)
C10—N2—N3—C8	0.0 (2)	C19—N2—C10—C7	−179.44 (19)
C6—C1—C2—C3	0.5 (4)	C10—N1—C11—C18	−1.4 (3)
C1—C2—C3—C4	1.3 (4)	C10—N1—C11—C12	178.00 (18)
C1—C2—C3—Cl1	−178.8 (2)	N1—C11—C12—C17	−10.3 (3)
C2—C3—C4—C5	−1.0 (4)	C18—C11—C12—C17	169.2 (2)
Cl1—C3—C4—C5	179.13 (19)	N1—C11—C12—C13	170.2 (2)
C3—C4—C5—C6	−1.1 (4)	C18—C11—C12—C13	−10.3 (3)
C3—C4—C5—Cl2	176.46 (19)	C17—C12—C13—C14	−0.3 (4)
C2—C1—C6—C5	−2.4 (4)	C11—C12—C13—C14	179.2 (2)
C2—C1—C6—C7	178.6 (2)	C12—C13—C14—C15	−0.8 (4)
C4—C5—C6—C1	2.7 (3)	C13—C14—C15—F4	−179.7 (3)
Cl2—C5—C6—C1	−174.81 (17)	C13—C14—C15—C16	1.4 (5)
C4—C5—C6—C7	−178.3 (2)	C14—C15—C16—C17	−0.9 (4)
Cl2—C5—C6—C7	4.2 (3)	F4—C15—C16—C17	−179.8 (2)
C1—C6—C7—C10	46.2 (3)	C15—C16—C17—C12	−0.2 (4)
C5—C6—C7—C10	−132.7 (2)	C13—C12—C17—C16	0.8 (4)
C1—C6—C7—C8	−125.9 (3)	C11—C12—C17—C16	−178.7 (2)
C5—C6—C7—C8	55.2 (3)	N1—C11—C18—C19	0.4 (3)
N2—N3—C8—C7	−0.4 (2)	C12—C11—C18—C19	−179.0 (2)
N2—N3—C8—C9	177.5 (2)	C11—C18—C19—N2	0.9 (3)
C10—C7—C8—N3	0.6 (3)	C11—C18—C19—C20	−176.8 (2)
C6—C7—C8—N3	173.9 (2)	N3—N2—C19—C18	179.1 (2)
C10—C7—C8—C9	−177.0 (2)	C10—N2—C19—C18	−1.1 (3)
C6—C7—C8—C9	−3.8 (4)	N3—N2—C19—C20	−3.2 (3)
C11—N1—C10—C7	−179.4 (2)	C10—N2—C19—C20	176.65 (19)
C11—N1—C10—N2	1.2 (3)	C18—C19—C20—F1	115.6 (2)
C8—C7—C10—N1	−180.0 (2)	N2—C19—C20—F1	−62.1 (3)
C6—C7—C10—N1	6.1 (4)	C18—C19—C20—F3	−3.9 (3)
C8—C7—C10—N2	−0.6 (2)	N2—C19—C20—F3	178.4 (2)
C6—C7—C10—N2	−174.44 (19)	C18—C19—C20—F2	−124.3 (2)
N3—N2—C10—N1	179.88 (19)	N2—C19—C20—F2	58.1 (3)
C19—N2—C10—N1	0.0 (3)		
